# Editorial: Alternative splicing in brain function

**DOI:** 10.3389/fnmol.2023.1335549

**Published:** 2023-11-23

**Authors:** Kif Liakath-Ali, Matthias Soller

**Affiliations:** ^1^Department of Molecular and Cellular Physiology, Stanford University, Stanford, CA, United States; ^2^School of Biological Sciences, University of Southampton, Southampton, United Kingdom; ^3^School of Biosciences, University of Birmingham, Birmingham, United Kingdom; ^4^Birmingham Centre for Genome Biology, University of Birmingham, Birmingham, United Kingdom

**Keywords:** RNA, brain, neuron, alternative splicing (AS), post-transcriptional regulation

Alternative splicing is a major mechanism to increase the number of proteins that can be made from the limited number of genes present in the human genome. During transcription of genes into precursor messenger RNA (pre-mRNA), non-coding introns are spliced out to make a messenger RNA (mRNA) that encodes the functional protein. During the splicing process some exons can be included or excluded and this process is termed alternative splicing. This is a highly regulated process that produces diverse mature mRNA transcripts from a single gene. Alternative splicing is present in almost every gene and is widespread in eukaryotic evolution. Moreover, the majority of genes expressed in the mammalian central nervous system undergo extensive alternative splicing, with some genes capable of contributing to over a thousand isoforms. This results in a variety of proteoforms exhibiting differences in function, binding preferences, catalytic activity, and localization. Disruptions in alternative splicing have been associated with numerous neurological disorders. A comprehensive understanding of its role in healthy and pathological nervous system function is still emerging. It is timely to gather current knowledge, advancement and challenges in this field. With this objective, we brought together several articles that discuss involvement of splicing and associated genetic perturbations in the central nervous system across the evolutionary scale—from fly to human.

Many RNA binding proteins (RBPs) play a crucial role in splicing regulation. The review by Feng et al. focuses on the heterogeneous nuclear ribonucleoprotein A1 (hnRNPA1), a key RBP associated with neurodegeneration and cancer. The authors discuss hnRNPA1's role in gene transcription, mRNA translation, and stability, highlighting its importance and potential as a therapeutic target. Another study by Titus et al. reveals the functional role of the RBP Caper in *Drosophila*, emphasizing its significance in sensory and motor neurons development and its regulatory role in *Drosophila* gravitaxis behavior.

Several studies identify splicing mutations associated with various neurological conditions. Lu Y. Q. et al. reveal the causative role of TANK-binding kinase (TBK1) in Amyotrophic Lateral Sclerosis (ALS) through mutational analysis, emphasizing the importance of intronic sequencing and pre-mRNA splicing analysis in understanding the complex mutational spectrum and pathogenesis of ALS. Reis et al. uncover a severe early onset dementia syndrome caused by an intronic splice donor variant in expanding our understanding of early onset dementia syndromes with a digenic background. Chen et al. identify a *de novo* splicing variant of the FOXP1 (Forkhead Box P1) gene in a patient with FOXP1 syndrome (an autosomal dominant neurodevelopmental disorder), providing insights into the genetic basis of global developmental delay, intellectual disability, and language delay. Fan et al. identify a disease-causing and aberrant splicing-inducing variant of TSC complex subunit 2 gene (TSC2) in a Han-Chinese family with Tuberous Sclerosis Complex (TSC), expanding the phenotypic and genetic spectrum of TSC and potentially contributing to its diagnosis and treatment. Wang et al. report a novel heterozygous STXBP1 (Syntaxin Binding Protein 1) splice variant with abnormal intron retention in a patient with Ohtahara syndrome (a rare form of epilepsy), highlighting the significance of splicing defect analysis in understanding the pathophysiology of neurodevelopmental disorders. Levchenko et al. reveal a deep intronic variant in the SNX14 (Sorting Nexin 14) gene in patients with spinocerebellar ataxia type 20, providing insights into the molecular pathogenic mechanism underlying the formation of a novel donor splicing site and potential therapeutic implications.

Tauopathies, including frontotemporal dementia and Alzheimer's disease (AD), are neurodegenerative diseases caused by tau brain aggregates. Tau protein, a microtubule-associated protein, can be disrupted in disease states due to the balance of tau splice isoforms. Xia et al. assess multiple mutations in three repeat (3R) tau for microtubule binding properties and prion-like aggregation propensity, contributing to the understanding of diverse presentations of tauopathies. Using bioinformatics pipelines, Farhadieh and Ghaedi reveal alternative splicing events (ASEs) in postmortem brain tissue with a cell-specific perspective, providing insights into AD pathology at the cell level. Lu Y. et al. identify several significant AS events in an AD mouse model, offering novel pathological mechanisms mediated by splice changes. Alalwany et al. investigate the neuroprotective effects of VEGF splice isoforms against AD-related neurotoxicity, suggesting potential therapeutic avenues.

Aging is a major risk factor for neurological disorders including dementia. Winsky-Sommerer et al. analyze the transcriptome and translatome in the female mouse hippocampus at different ages, revealing age-associated splicing changes and their potential role in age-related deficits in hippocampal-dependent behavior. The study provides a comprehensive resource for understanding age-associated splicing changes with implications for neurological diseases.

Differential splicing of exons in neurons can alter protein properties, including ion channels, neurotransmitter receptors, and synaptic cell adhesion molecules. Baxter et al. explore the correlation between high K+ exposure, delayed-onset NMDA receptor-dependent neuronal death, and exon inclusion levels in neurons and astrocytes *in vitro*. The study highlights the neurotoxic nature of certain stimulation paradigms and emphasizes the importance of NMDA receptor blockade.

The Research Topic brings together up-to-date research focused on the biology of splicing and its regulators and associated mutations in neurological diseases. It provides new insights into the pathophysiological role of splicing modulations and offers possible strategies for therapeutic targets.

## Author contributions

KL-A: Conceptualization, Writing – original draft, Writing – review & editing. MS: Conceptualization, Writing – original draft, Writing – review & editing.

